# Analysis of Gait Motion Changes by Intervention Using Robot Suit Hybrid Assistive Limb (HAL) in Myelopathy Patients After Decompression Surgery for Ossification of Posterior Longitudinal Ligament

**DOI:** 10.3389/fnbot.2021.650118

**Published:** 2021-03-31

**Authors:** Seioh Ezaki, Hideki Kadone, Shigeki Kubota, Tetsuya Abe, Yukiyo Shimizu, Chun Kwang Tan, Kousei Miura, Yasushi Hada, Yoshiyuki Sankai, Masao Koda, Kenji Suzuki, Masashi Yamazaki

**Affiliations:** ^1^Department of Orthopaedic Surgery, University of Tsukuba, Tsukuba, Japan; ^2^Center for Cybernics Research, University of Tsukuba, Tsukuba, Japan; ^3^Department of Rehabilitation Medicine, University of Tsukuba, Tsukuba, Japan; ^4^Faculty of Engineering, Information and Systems, University of Tsukuba, Tsukuba, Japan

**Keywords:** hybrid assistive limb, ossification of the posterior longitudinal ligament, rehabilitation, postoperative procedures, robotics, gait analysis

## Abstract

Ossification of the posterior longitudinal ligament (OPLL) is a hyperostonic condition in which the posterior longitudinal ligament becomes thick and loses its flexibility, resulting in ectopic ossification and severe neurologic deficit (Matsunaga and Sakou, 2012). It commonly presents with myelopathy and radiculopathy and with myelopathy progression motor disorders and balance disorders can appear. Even after appropriate surgical decompression, some motor impairments often remain. The Hybrid Assistive Limb (HAL) is a wearable powered suit designed to assist and support the user's voluntary control of hip and knee joint motion by detecting bioelectric signals from the skin surface and force/pressure sensors in the shoes during movement. In the current study, the HAL intervention was applied to 15 patients diagnosed with OPLL who presented with myelopathy after decompression surgery (6 acute and 9 chronic stage). Following the HAL intervention, there were significant improvements in gait speed, cadence, stride length, in both acute and chronic groups. Joint angle analysis of the lower limbs showed that range of motion (ROM) of hip and knee joints in acute group, and also ROM of hip joint and toe-lift during swing in chronic group increased significantly. ROM of knee joint became closer to healthy gait in both groups. Electromyography analysis showed that hamstrings activity in the late swing phase increased significantly for acute patients. Immidiate effect from HAL session was also observed. EMG of vastus medialis were decreased except chronic 7th session and EMG of gastrocnemius were decreased except acute 7th session, which suggests the patients were learning to walk with lesser knee-hypertension during the sessions. After all, double knee action appeared in both acute and chronic groups after the HAL intervention, rather than knee hyper-extension which is a common gait impairment in OPLL. We consider that these improvements lead to a smoother and healthier gait motion.

## 1. Introduction

Myelopathy refers to a pathology that causes neurological deficits in the spinal cord (Oyinkan Marquis and Capone, 2016), that usually results from compression of the spinal cord by osteophytes or extruded intervertebral disks between the cervical spine (Seidenwurm, 2008). Age-related degeneration of the tissues in the spinal column, known as degenerative myelopathy, is the most common cause of spinal cord dysfunction among older adults. The structural changes include: degeneration of intervertebral disc, vertebral bodies, and facet joints; hypertrophy of the ligament flavum; and ossification of the posterior longitudinal ligament (OPLL) (Fehlings et al., 2017). OPLL is a hyperostonic condition characterized by pathological ectopic ligament ossification that mostly affects the cervical or thoracic spine segments (Epstein, 2002; Matsunaga and Sakou, 2012) and commonly presents with myelopathy and radiculopathy. OPLL was previously considered specific to Asian population (Mizuno and Nakagawa, 2006), and the Japanese have a significantly high incidence rate of this disease (Matsunaga and Sakou, 2006); however, its prevalence among Caucasians has been increasing recently (Fehlings et al., 2015). Motor disorders and balance disorders can appear with myelopathy progression. Spinal stenosis syndrome, which largely stems from motor weakness, can occur during or after middle age (McKinley et al., 1998). When symptoms are mild, observation, physical therapy, and oral analgesics are chosen as a means of non-operative management. However, when myelopathy progresses or is irresponsive to conservative management, surgical management is usually adopted. Surgery is thought to be an effective treatment for patients with myelopathy or severe stenosis (Epstein, 2014). However, even after appropriate decompression operation has been successfully achieved, patients are sometimes left with residual motor impairments. The long-term postoperative prognosis tends to be a gradual re-worsening of neurological symptoms after 5 years (Committee for Clinical Guidelines of the Japanese Orthopaedic Association, 2011). Several methods for gait rehabilitation are available for patients with myelopathy following surgical decompression including muscle training, range of motion training, movement equilibrium training, and sphincter control. Recently, there has been a lot of discussion about the potentials and limitations of treadmill-based rehabilitation. Several studies have shown that there are no advantages to using passive robotic rehabilitation such as treadmill walking (Hidler et al., 2009) compared to conventional rehabilitation therapy (Dobkin et al., 2006).

Recent studies have reported the use of the exoskeleton robot suit, the Hybrid Assistive Limb (HAL) (Cyberdyne, Tsukuba, Japan) as a novel approach to manage gait rehabilitation (Sankai and Sakurai, 2018). HAL is a wearable powered suit, designed to assist and support users' voluntary control of hip and knee joint motion by detecting bio-electric signals (muscle action potentials) from surface electrodes and from force/pressure sensors in the shoes (Kawamoto and Sankai, 2005; Sankai and Sakurai, 2018). Compared to other robot training, HAL can enhance the wearer's voluntary motion, and intensify its feedback. Recent studies have reported the feasibility and effectiveness of HAL for functional recovery in rehabilitation use for multiple neurological disorders patients with gait disturbance after spinal cord diseases and cerebrovascular diseases such as chronic stroke (Kawamoto et al., 2013; Nilsson et al., 2014; Wall et al., 2015; Kasai and Takeda, 2016), chronic spinal cord injury (Kubota et al., 2013; Aach et al., 2014; Sczesny-Kaiser et al., 2015; Wall et al., 2015; Ikumi et al., 2017), and OPLL (Sakakima et al., 2013; Kubota et al., 2016, 2019; Fujii et al., 2017; Puentes et al., 2018). For the chronic stage of myelopathy in patients with postoperative cervical OPLL, improvement in gait speed, step length, and cadence has been observed (Kubota et al., 2016). Furthermore, it has enabled patients to use the knee extensor in the stance phase without locking the knee by supporting gluteus maximus and quadriceps muscle activation (Shimizu et al., 2017, 2018).

The purpose of rehabilitation robotics is not to replace human therapists, but to provide a tool to increase productivity (Tejima, 2001) and deliver appropriate rehabilitation to more patients. This technology increases the repeatability of movement and also provides tools to assess the progress and effectiveness of the rehabilitation processes (Huang and Krakauer, 2009).

Also, achieving a faster gait speed through rehabilitation should be done while ensuring that no compensational gait is developed (Verma et al., 2012). If unhealthy gait such as hyper knee extension develops, it is more likely to result in locomotive syndrome or the need for further surgical intervention (Bleyenheuft et al., 2010; Mao et al., 2015). As the population is aging and the life expectancy after surgery is lengthening, there is an increasing need for maintaining a good quality of life through the development of a healthier gait.

In this study, we analyse the joint angles and muscle activities during gait before and after HAL gait intervention, and report changes therein and also improvements of double knee action and walking ability, in 15 patients with postoperative OPLL (6 acute and 9 chronic stage) who presented myelopathic gait in their acute and the chronic stage after decompression surgery.

## 2. Methods

### 2.1. Participants

Fifteen patients who had undergone decompression surgery after diagnosed with OPLL associated with severe motor impairment (Table 1). Six of them (3 male and 3 female) received the HAL intervention at an acute stage of postoperative gait disorder. The mean age of the patients at an acute stage was 57.8 years old, and HAL intervention was started at a mean of 25 days after surgery. The other nine patients (9 males) received the intervention at a chronic stage of postoperative gait disorder. The mean age of the patients at a chronic stage was 68.3 years old and HAL intervention was started at a mean of 1023.2 days after surgery. Kinematic data from eight healthy volunteers (3 male, 5 female) who did not receive HAL treatment was used for comparison as a healthy gait. The mean age of healthy volunteers was 57 years old.

**Table 1:** Subject characteristics.

**Participant ID**	**Group**	**Sex**	**Age**	**Height (cm)**	**Weight (kg)**	**Surgery-HAL interval (days)**
A1	Acute	F	78	146	51	15
A2	Acute	M	64	165	90	26
A3	Acute	M	52	180	100	18
A4	Acute	F	63	154	56	32
A5	Acute	F	41	156	79	31
A6	Acute	M	49	169	80	28
C1	Chronic	M	70	168	71	288
C2	Chronic	M	75	168	78	287
C3	Chronic	M	68	174	64	3655
C4	Chronic	M	78	159	61	372
C5	Chronic	M	76	166	63	2188
C6	Chronic	M	58	176	72	540
C7	Chronic	M	66	157	74	730
C8	Chronic	M	70	163	69	958
C9	Chronic	M	44	174	100	191
H1	Healthy	F	56	157	59	-
H2	Healthy	F	42	161	49	-
H3	Healthy	F	59	152	53	-
H4	Healthy	F	67	164	50	-
H5	Healthy	F	60	158	57	-
H6	Healthy	M	50	163	58	-
H7	Healthy	M	45	164	61	-
H8	Healthy	M	77	170	59	-
H9	Healthy	M	66	166	62	-

*Surgery-HAL interval refers to the number of days elapsed from the surgery to the beginning of HAL therapy*.

Each of the 15 patients was provided with 10 HAL gait rehabilitation intervention sessions. Each HAL session lasted approximately 1 h, which included 20 min of HAL-assisted walking. The HAL assist parameters were configured to the patient's comfort for each session. The All-in-One walking device (Ropox Inc., Denmark) was used alongside the HAL. When necessary, weight support was provided for the acute patients so that they could walk by themselves. Signed informed consent was obtained from each participant after they received a full explanation about the present research and its data usage. This study was approved by the University of Tsukuba Hospital Ethics Committee (Approval number: H26-22).

### 2.2. HAL Set up

The double-legged version of the HAL suit was used for this study. This HAL suit has a total of four electric motors, located bilaterally at the patient's hip and knee joints, which assist the wearer's leg motion and gait. The hip and knee motors were actuated for producing torque in proportion to a weighted difference of the respective muscles. Activation of these motors is guided in real time by neuromuscular activities detected by surface electrodes placed at the iliopsoas (hip flexor), gluteus maximus (hip extensor), biceps femoris (knee flexor), and quadriceps (vastus lateralis and knee extensor).

### 2.3. HAL Intervention

The HAL intervention included 10 sessions of HAL therapy conducted twice per week at 90 min per session. Patients at an acute stage of disease progression began HAL therapy during their hospitalization period, while those at a chronic stage began as outpatients. Each session began with fitting the HAL suit. For those who needed, a walking device (All-in-One Walking Trainer, Ropox A/S, Naestved, Denmark) with a harness was provided to support body weight and prevent falling. The HAL therapy session consisted of 20 min of walking at a comfortable pace on a 25-m oval-shaped walking course with rest intervals. Vital signs including blood pressure, heart rate, and oxygen saturation were recorded at the beginning, during, and end of each session to ensure that patients were stable. The patients' lower limb joint control ability was assessed by a clinician before and after the HAL intervention using MMT (Manual muscle testing) scale grading 0–5.

### 2.4. Gait Measurement

#### 2.4.1. Joint Angles

The functional evaluation and the walk tests were carried out without the participant wearing the HAL suit before the first session and after the last one. The time and the number of steps were counted while they walked 10 m in a straight line at a comfortable pace, and the speed and stride length were calculated using this data. Gait before and after the HAL intervention was measured and compared using a motion capture system (VICON MX System, 16 T20s cameras, 100 Hz, Plug-in gait marker set, Oxford, UK), which was synchronized with EMG and sampled at 100 Hz. Sixteen auto-reflective makers were placed in accordance with the plug-in gait marker set bilaterally on the anterior superior iliac spine, posterior superior iliac spine, lower lateral 1/3 surface of the thigh, the flexion-extension axis of the knee, lower lateral 1/3 surface of the shank, lateral malleolus of the ankle, the posterior peak of the calcaneus for the heel, and the lateral second metatarsal bone of the toe. Sagittal angles of bilateral hip, knee and ankle joints were extracted using VICON Nexus software (version 2.2.3). In the pipeline processing of marker position data, the software applies a Woltering filter (Woltring, 1986) which is based on spline fitting for smoothing the marker trajectories.

#### 2.4.2. Electromyography

Electromyography was recorded during 10 m test before the first HAL session and after the last HAL session for pre-post comparison, and also in the 4th and 7th HAL sessions immediately before and after using HAL. The 4th session represents the first half of the ten sessions, and the 7th session was extracted as information representative of the second half of the ten sessions.

Six wireless, surface EMG electrodes were placed bilaterally on the muscles relevant to knee joint motion; vastus medialis (VM), hamstrings (HAM), and gastrocnemius (GAS) for the purpose of evaluating knee joint control during gait. The data was sampled using a TrignoTM Lab Wireless electromyography system (Delsys Inc., Boston, MA, USA) at 2k Hz synchronized with the motion capture.

### 2.5. Data Analysis

#### 2.5.1. Joint Angles

Data on walking speed, cadence and stride length were obtained from clinical assessment. From the motion capture data, joint angle profile of hip, knee and ankle joints were obtained and segmented into step cycles referring to the gait phase detected by toe and heel markers. Swing time and stance time, swing time ratio were obtained. For joint angle analysis, the maximum extension and flexion of hip, knee, ankle joint angles within cycle, and range of motion (ROM) of these joint angles were obtained. The anterior and posterior tilt angles of the pelvis, and toe-lift from the ground before and after the intervention were also compared. Knee joint angle is known to possess double flexion peaks in a gait cycle; one during the stance phase and the other during the swing (Perry and Burnfield, 2010). The first peak after heel landing corresponds to the shock absorption and the second peak corresponds to the flexion during swing phase. The amount of the first flexion was evaluated by the difference between the knee joint angles at the heel landing and the first flexion peak, which is hereafter referred to as the first knee action.

#### 2.5.2. Electromyography

Each patient's steps, from first heel-strike to final toe-off were extracted for each leg, and the synchronized electromyography (EMG) tracks and motion data were collected. Hampel filter (time window = 200; σ threshold = 4 [standard deviations]) was used to remove artifacts from the EMG data, before being rectified and low-passed filtered (4th order zero-lag Butterworth low-pass filter at 6 Hz to obtain the EMG envelope and then time-normalized and re-sampled to 100-time points.

Regarding muscle activities related to the control of knee joint during gait, deceleration of the angle is known to take place during late swing to prepare for the coming heel strike and the first knee action (Perry and Burnfield, 2010). This phase is characterized as the range between 80 and 100% of a gait cycle (Levangie and Norkin, 2005) including terminal swing which starts when the tibia crosses the vertical position and ends with the initial contact. The gait phases were extracted referring to the motion capture data which was recorded in synchronization with the EMG. The ratio of EMG values in the 80–100% of gait cycle to those in the entire cycle was calculated and compared between pre and post HAL intervention. For evaluation of immediate EMG changes in the 4th and 7th sessions, integrated EMG values in the 80–100% of gait cycle was calculated and compared between just before and after HAL intervention within each of the sessions.

### 2.6. Statistical Analysis

All data are expressed with mean and standard deviation (SD). Comparisons of kinematic and kinetic data between before and after the HAL intervention were made using a paired Wilcoxon signed-rank test for the acute and chronic groups, to compare between baseline measurements and outcomes after all the 10 sessions. Mann–Whitney *U*-Test was used to compare MMT scores between baseline and outcomes. Significance was considered in comparisons with *P* < 0.05.

### 2.7. Software Tools

Custom scripts on MATLAB 8.4 and 9.3 (Mathworks Inc., Natick, MA, USA) and R 3.6.0 (The R Foundation for Statistical Computing) were used for data extraction and statistical analysis.

## 3. Results

### 3.1. HAL Intervention for Patients at an Acute Stage of Postoperative Gait Disorder

**Figure 1:**
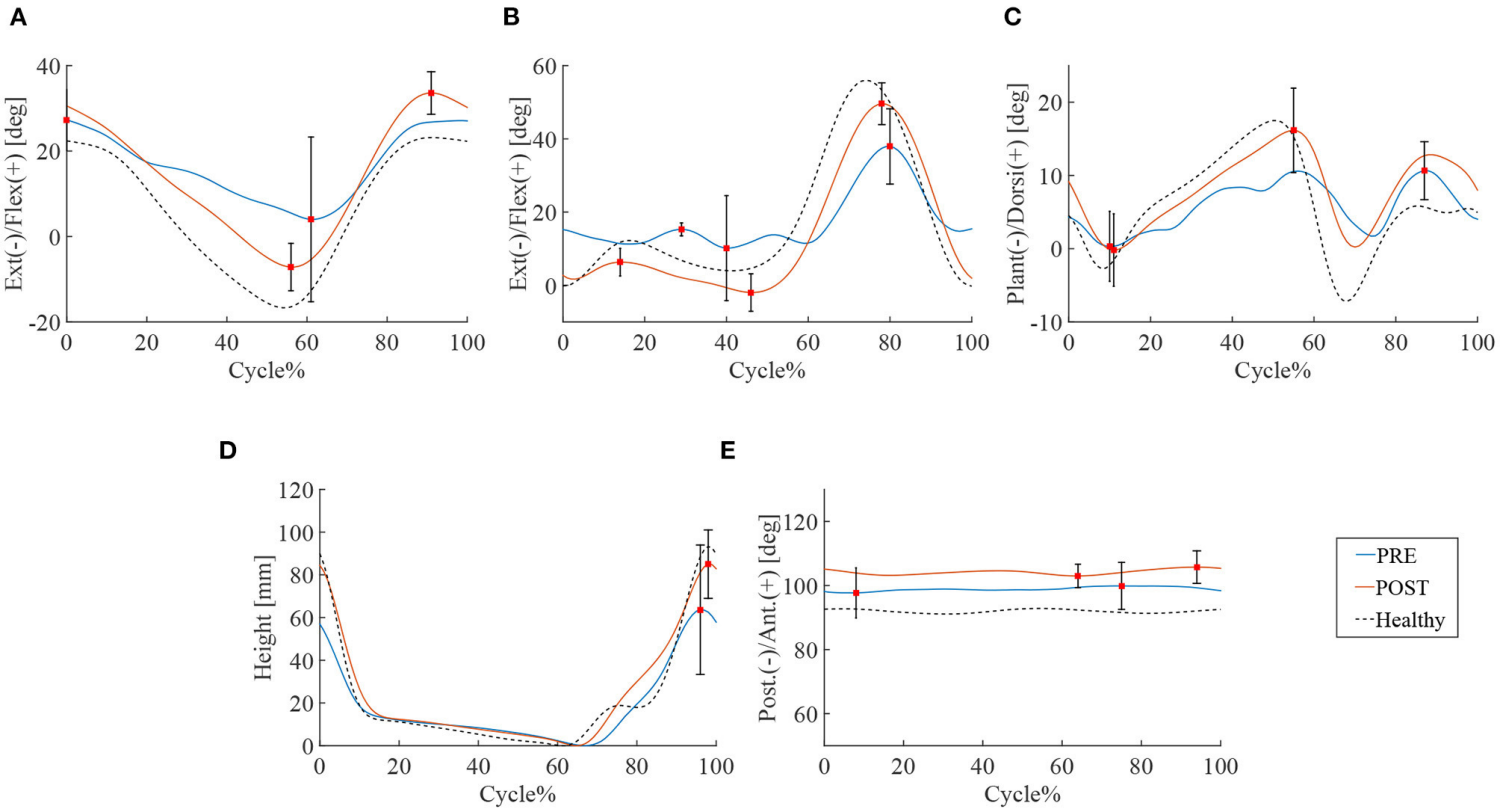
Gait kinematics of acute group patients. Blue line is the mean of patients' joint angle before HAL intervention, the red line is post HAL intervention, and the dotted line is gait analysis plot for healthy subjects. The percentage of walking cycle represents the normalized time. **(A)** Hip joint. **(B)** Knee joint. **(C)** Ankle joint. **(D)** Toe lift. **(E)** Pelvis.

14.2 ± 5.7 steps and 10.8 ± 2.0 steps were recorded by the motion capture in 10 m walking tests before and after the HAL intervention, respectively. Significant improvements were observed in walking speed 24.5 vs. 54.1 m/min (*P* < 0.01), and stride length 71.2 vs. 102.5 cm (*P* < 0.05). Swing time and cadence tended to improve, respectively, by 1.1 vs. 0.8 s and cadence 35.7 vs. 50.7 cycles/min but statistical significance was not achieved (N.S.) (Table 2). A comparison of the peak of each leg joint angle was used to evaluate the limb movement during the gait (Figure 1). Measurements of hip joint range of movement (ROM) before and after HAL intervention indicated a significant increase in ROM: 34.0° vs. 43.4° (*P* < 0.05) (Figure 1A). Knee joint ROM also increased significantly: 42.1° vs. 56.7° (*P* < 0.05). The first knee action appeared in the initial stance stage: 3.2° vs. 5.5° (*P* < 0.05) (Figure 1B). Ankle joints ROM was 19.9° vs. 25.5° (N.S.) (Figure 1C) and the toe-lift was 132.2 vs. 144.8 mm (N.S.) (Figure 1D). MMT scores of the acute patients showed improvement in hip flexor (*P* < 0.01), knee extensor (*P* < 0.05), and ankle dorsi flexor (*P* < 0.05) muscle groups, but not in knee flexor and ankle plantor flexor muscle groups (Table 3).

**Table 2:** Comparison of gait kinematics between pre and post HAL intervention in acute stage patients.

**Measurements**	**Pre HAL**	**Post HAL**	**P-value**
Walking speed (meters/minute)	24.5	54.1	*P* < 0.01
Cadence (cycles/minute)	35.7	50.7	N.S.
Stride length (cm)	71.2	102.5	*P* < 0.05
Swing time (seconds)	1.1	0.8	N.S.
ROM of hip joints	34.0°	43.4°	*P* < 0.05
ROM of knee joints	42.1°	56.7°	*P* < 0.05
The first knee action	3.2°	5.5°	*P* < 0.05
ROM of ankle joints	19.9°	25.5°	N.S.
Toe lift (mm)	132.2	144.8	N.S.

**Table 3:** Comparison of MMT scores between pre and post HAL intervention in acute stage patients.

**Muscle group**	**Pre HAL**	**Post HAL**	**P-value**
	**Med [Min-Max]**	**Med [Min-Max]**	
Hip Flex	3 [3–4]	4 [3–5]	*P* < 0.01
Knee Flex	3 [3–4]	4 [3–4]	N.S.
Knee Ext	3.5 [3–5]	4 [4–5]	*P* < 0.05
Ankle Plant	3.5 [2–4]	4 [3–4]	N.S.
Ankle Dorsi	4 [3–4]	4 [3–5]	*P* < 0.05

### 3.2. HAL Intervention for Patients at a Chronic Stage of Postoperative Gait Disorder

**Figure 2:**
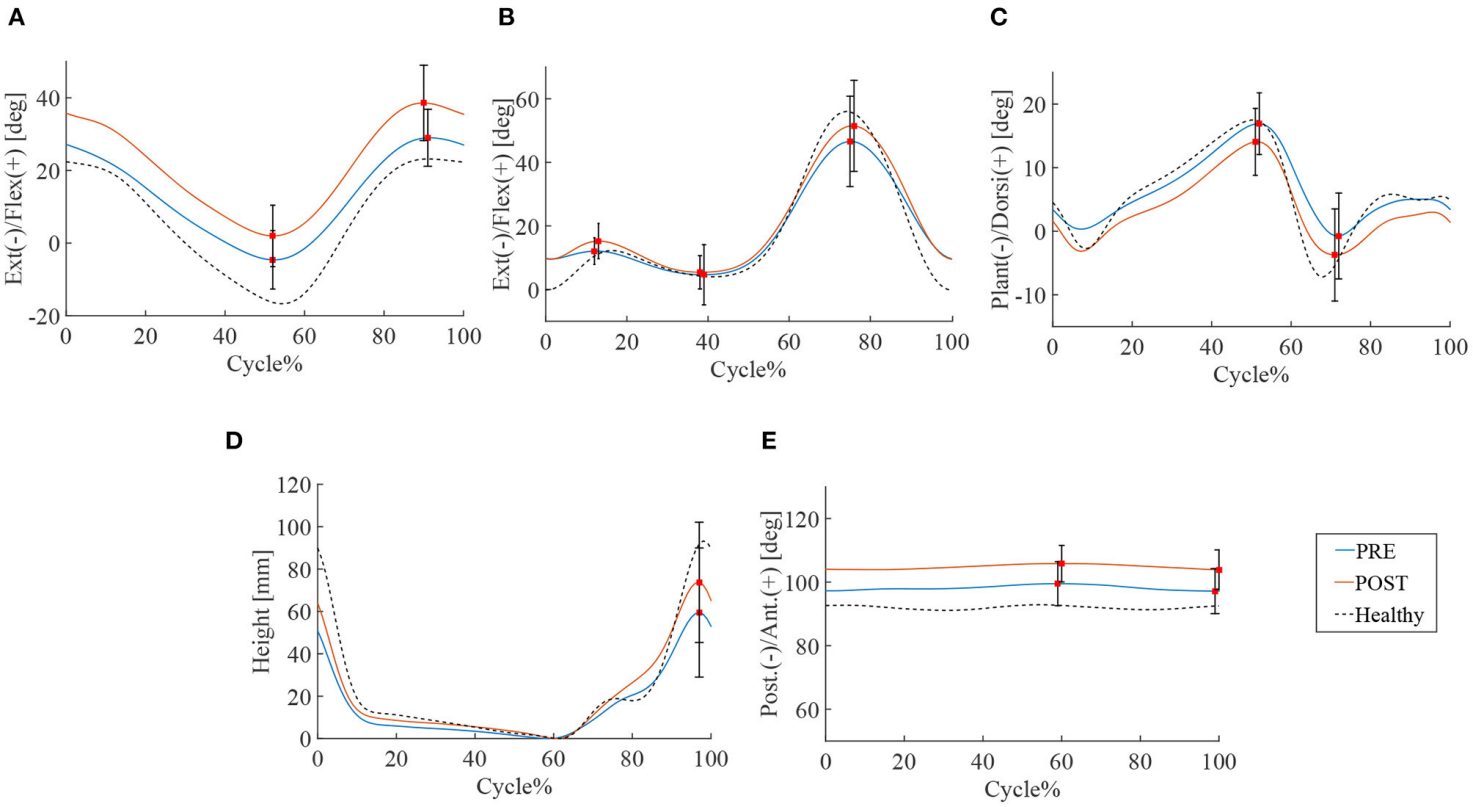
Gait kinematics of chronic stage patients. Blue line is the mean of patients' joint angle before HAL intervention, the red line is post HAL intervention, and the dotted line is gait analysis plot for percentage of walking cycle represents the normalized time. **(A)** Hip joint. **(B)** Knee joint. **(C)** Ankle joint. **(D)** Toe lift. **(E)** Pelvis.

16.0 ± 3.9 steps and 14.0 ± 3.5 steps were recorded by the motion capture in 10 m walking tests before and after the HAL intervention, respectively. Significant improvements were observed in walking speed 46.5 vs. 53.8 m/min (*P* < 0.01), cadence 51.7 vs. 54.7 cycles/min (*P* < 0.05), stride length 89.3 vs. 98.0 cm (*P* < 0.01), and swing time 0.91 vs. 0.92 s (*P* < 0.05) (Table 4). ROM of hip joint: 36.1° vs. 40.3° (*P* < 0.01) has increased and slightly changed to flexion position (Figure 2A). Knee joints ROM was non-significant, and the double knee action has appeared in the initial stance stage: 4.8° vs. 7.2° (*P* < 0.05) (Figure 2B). Ankle joints ROM was 25.5° vs. 26.7° (N.S.) (Figure 2C), and it changed to plantar flexion position. This change could have been caused by the displacement of the ROM of hip joint that moved to flexion position. Improvement in toe lift: 109.7 vs. 128.0mm (*P* < 0.01) was observed (Figure 2D). Pelvic angle has changed to an anterior tilt angle of 100.4° vs. 107.2° (*P* < 0.05) and a posterior tilt angle of 96.0° vs. 102.5° (*P* < 0.05) (Figure 2E). MMT scores of the chronic patients showed improvement in hip flexor muscle group (*P* < 0.05), but not in the other lower limb muscle groups (Table 5).

**Table 4:** Comparison of gait kinematics between pre and post HAL intervention in chronic stage patients.

**Measurements**	**Pre HAL**	**Post HAL**	**P value**
Walking speed (meters/minute)	46.5	53.8	*P* < 0.01
Cadence (steps/minute)	51.7	54.7	*P* < 0.05
Stride length (cm)	89.3	98.0	*P* < 0.01
Swing time (seconds)	0.91	0.92	*P* < 0.05
ROM of hip joints	36.1°	40.3°	*P* < 0.05
ROM of knee joints	46.9°	51.9°	N.S.
The first knee action	4.8°	7.2°	*P* < 0.05
ROM of ankle joints	25.5°	26.7°	N.S.
Toe lift (mm)	109.7	128.0	*P* < 0.01

**Table 5:** Comparison of MMT scores between pre and post HAL intervention in chronic stage patients.

**Muscle Group**	**Pre HAL**	**Post HAL**	**P value**
	**Med [Min-Max]**	**Med [Min-Max]**	
Hip Flex	4 [3–5]	4 [3–5]	*P* < 0.05
Knee Flex	4 [3–5]	5 [3–5]	N.S.
Knee Ext	4 [4–5]	5 [3–5]	N.S.
Ankle Plant	4 [2–5]	4 [2–5]	N.S.
Ankle Dorsi	4 [1–5]	5 [1–5]	N.S.

### 3.3. Comparison of Joint Angles With Healthy Subjects

**Figure 3:**
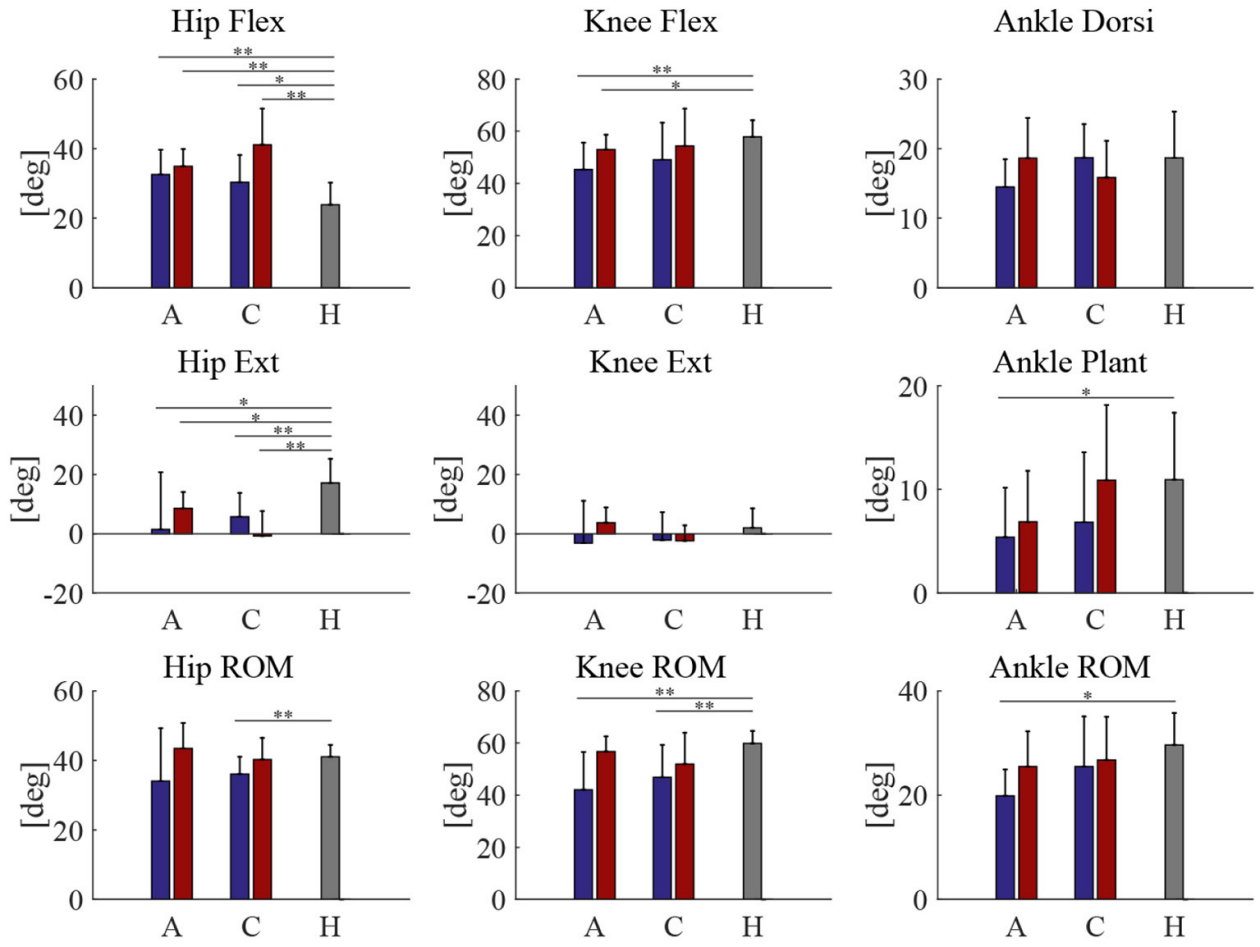
Peak joint angles | A, C, H is stands for acute, chronic, and healthy, respectively. Blue and red bars represent pre and post HAL intervention, respectively. **P < 0.01, *P < 0.05, and no sign means *P* > 0.05.

Peak joint angles of acute and chronic stage patients, both pre and post HAL intervention, was compared with healthy volunteers (Figure 3). Range of motion (ROM) of knee joint showed a significant difference between patients of both acute and chronic groups and healthy subjects before HAL intervention. However, these differences have disappeared after the HAL intervention in both acute and chronic phases (Figure 3, Knee ROM). Table 6 shows RMS (root mean squared) difference of gait kinematics parameters of acute and chronic stage patients, pre and post HAL intervention, in comparison to healthy subjects. It also shows the change of RMS between pre and post.

**Table 6:** RMS (root mean squared) difference of gait kinematics of acute and chronic stage patients, pre and post intervention, compared to healthy subjects, and its changes between pre and post.

		**Acute**			**Chronic**	
**Parameters**	**Pre**	**Post**	**Pre - post**	**Pre**	**Post**	**Pre - post**
Walking Speed (m/min)	43.0	18.6	**24.4**	23.6	18.7	**4.93**
Cadence (cycles/m)	17.8	8.6	**9.2**	7.1	7.3	−0.24
Stride Length (cm)	62.0	26.7	**35.4**	40.0	33.4	**6.53**
Swing Ratio (%)	14.9	12.0	**2.90**	8.99	3.56	**5.43**
Swing Duration (s)	0.24	0.11	**0.14**	0.074	0.11	−0.038
Stance Duration (s)	0.437	0.253	**0.184**	0.155	0.0725	**0.0825**
Hip Max Flex (deg)	11.0	12.0	−0.968	9.94	19.9	−9.97
Hip Max Ext (deg)	24.1	10.1	**14.0**	13.8	19.8	−5.93
Hip ROM (deg)	16.1	7.37	**8.69**	6.91	5.97	**0.935**
Knee Max Flex (deg)	15.9	7.30	**8.58**	16.3	14.2	**2.03**
Knee Max Ext (deg)	14.6	5.17	**9.38**	10.0	6.71	**3.34**
Knee ROM (deg)	22.4	6.33	**16.08**	17.7	14.0	**3.64**
Ankle Max Dors (deg)	5.62	5.47	**0.149**	4.67	5.80	−1.12
Ankle Max Plant (deg)	7.16	6.24	**0.925**	7.70	6.98	**0.717**
Ankle ROM (deg)	10.8	7.62	**3.22**	10.1	8.47	**1.66**
Toe Max Height (mm)	30.6	15.3	**15.2**	44.1	31.1	**13.1**
Pelvis Max Ant. (deg)	9.99	13.6	−3.66	9.34	14.6	−5.23
Pelvis Max Post. (deg)	9.21	12.3	−3.07	8.62	13.5	−4.83
Pelvis ROM (deg)	2.52	2.20	**0.328**	2.40	2.20	**0.195**
Double Knee Action (deg)	9.81	8.18	**1.63**	8.99	7.74	**1.25**

*Bold numbers indicate positive pre-post RMS, meaning closer to healthy values at post HAL assessment than at pre HAL*.

### 3.4. EMG Analysis of Double Knee Action

**Figure 4:**
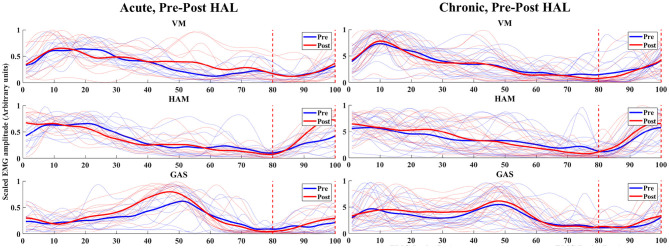
EMG (VM, HAM, and GAS) of acute and chronic stage patients, pre and post HAL intervention – The normalized time correspond to the percentage of walking cycle. The blue and red lines shows pre and post HAL intervention plots of each subject's EMG, respectively. The solid blue and red lines are mean plots of all subject's pre and post HAL intervention in the group, respectively. Eighty percent to one hundred percent of gait cycle and the late swing phase to terminal swing phase was estimated using synchronized motion data.

For acute patients, the ratio of EMG values of HAM in the late to terminal swing phase (80–100% of normalized gait cycle) against the full cycle was significantly higher in post-HAL intervention (*P* = 0.0137) (Figure 4, Acute). However, EMG values of VM (*P* = 0.3804) and GAS (*P* = 0.5693) were not significantly different. Chronic patients did not show significant differences; VM (*P* = 0.1330), HAM (*P* = 0.6791) and GAS (*P* = 0.6165) (Figure 4, Chronic).

### 3.5. Analysis of Immediate Muscle Activity Changes

**Figure 5:**
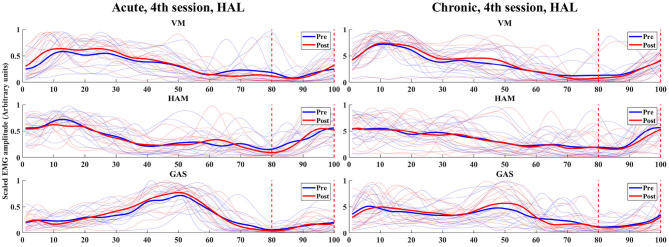
EMG (VM, HAM, and GAS) of acute and chronic stage patients, pre and post 4th HAL session.

**Figure 6:**
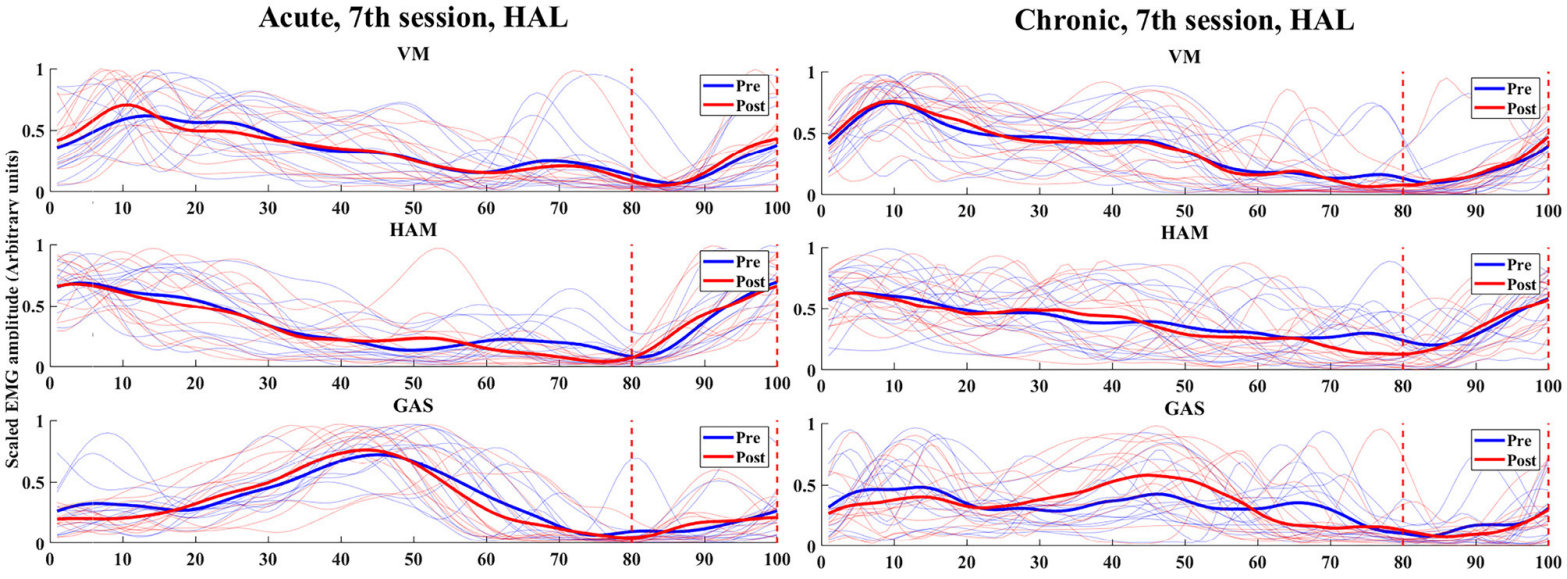
EMG (VM, HAM, and GAS) of acute and chronic stage patients, pre and post 7th HAL session.

In the analysis of immediate gait changes in the 4th and 7th sessions (Figures 5, 6 and Tables 7, 8), walking speed, cadence, stride length or toe lift did not show significant difference in both of acute and chronic stage patients, except that only toe lift became higher at the 4th session in the chronic stage patients (*P* < 0.05). On the other hand, EMG analysis of late to terminal swing phase showed that activation of VM decreased in the 4th HAL session of the acute patients (*P* < 0.01) and also tended to decrease at the 7th session of the acute and at the 4th session of chronic patients. (N.S., 4th Chronic *P* = 0.091, 7th Acute *P* = 0.077). Activation of GAS decreased in the 7th session of the chronic patients (*P* < 0.01) and tended to decrease at the 4th HAL session of both acute and chronic patients (N.S., 4th Acute *P* = 0.064, 4th Chronic *P* = 0.058) (Tables 5, 6).

**Table 7:** Comparison of integrated EMG of the late to terminal swing phase during 10m walking test immediately before and after HAL intervention at the 4th HAL session, in acute and chronic stage patients.

**4th session**		**Acute**			**Chronic**	
**Parameters**	**Pre**	**Post**	**P-value**	**Pre**	**Post**	**P-value**
Walking speed (m/min)	40.2	40.1	0.964	42.9	42.8	0.936
Cadence (cycles/min)	41.0	42.5	0.114	47.9	45.7	0.380
Stride length (cm)	84.6	84.1	0.833	75.4	68.6	0.672
Toe lift (mm)	93.8	99.3	0.239	76.5	82.7	**0.013**
VM (mV)	0.1743	0.1169	**0.000**	0.3759	0.2290	0.091
HAM (mV)	0.2457	0.2144	0.380	0.2253	0.1858	0.469
GAS (mV)	0.3000	0.1087	0.064	0.2082	0.1630	0.058

*Bold numbers mean P < 0.05 or P < 0.01*.

**Table 8:** Comparison of integrated EMG of late to terminal swing phase during 10m walking test immediately before and after HAL intervention at the 7th HAL session, in acute and chronic stage patients.

**7th session**		**Acute**			**Chronic**	
**Parameters**	**Pre**	**Post**	**P value**	**Pre**	**Post**	**P value**
walking speed (m/min)	53.8	51.3	0.101	43.5	40.1	0.378
cadence (cycles/min)	49.5	47.5	0.053	45.2	46.2	0.289
stride length (cm)	92.7	93.9	0.584	83.5	82.5	0.646
toe lift (mm)	105.5	105.7	0.984	82.9	86.9	0.294
VM (mV)	0.1694	0.1305	0.077	0.9187	0.2837	0.268
HAM (mV)	0.1678	0.1711	0.791	0.2705	0.2446	0.578
GAS (mV)	0.1363	0.1123	0.176	0.3191	0.2139	**0.009**

*Bold numbers mean that P < 0.05 or P < 0.01*.

## 4. Discussion

Improvements were observed in the form of faster walking speed, higher cadence and longer strides with smooth foot clearance and shortened swing time in both acute and chronic phase patients. Increased ROM of hip and knee joints contributed to longer strides. First knee action which is typical for a healthy gait has appeared after the HAL intervention, enabling the patients to use knee as a cushion at initial contact. Even without the significant changes in ankle joint, there were improvement in toe lift. It was achieved by the combination of enhanced ROM of hip and knee joints. In chronic patients, ROM of the hip joint slightly shifted to flexion position, while the ankle joint's ROM shifted to plantar flexion position. Regarding the toe lift, the geometrical relationship is that flexion of the hip improves the toe lift, while plantar flexion of the ankle decreases the toe lift. Since the data shows that the toe lift improved after HAL intervention, the combination of these two shifting resulted in higher toe lifting. This is beneficial in daily practical walking, in that it contributes to prevent stumbling. When the patients walked with HAL, we observed that they are more likely to walk with their legs up high. Chronic patients who were able to walk in the first stage were especially prone to gait accentuation, and this series of improvements in ROM made them raise their toes higher.

Anterior shift of the pelvic orientation was observed in both the acute and the chronic patients. We consider that this was caused by the HAL's mechanical structure, which tends to constrain pelvic posture. With respect to angular momentum, pelvic rotation and its stability is important in high velocity walking rather than in slower walking (below 3 km/h), since legs and arms play more important roles in faster walking (Bruijn et al., 2008). So that this slight pelvic angle change might not be a problem in low velocity walk during rehabilitation. It was also discussed that knee flexion amount contributes to controlling the center of mass more than pelvic rotation (Della Croce et al., 2001; Kerrigan et al., 2001). Therefore, we believe that the influence of pelvic rotation was small compared to the merit of gaining double knee action. It is left for future studies to improve the pelvis harness to achieve some level of rotational movement while providing enough support.

In a healthy gait, there are two episodes of knee flexion during gait; the first knee flexion takes place at the initial contact to loading response phase and the second knee flexion takes place during the period from terminal stance to pre-swing phase. This double knee action is important for a healthy gait, especially the first knee action is essential for shock absorption just after landing (Farrokhi et al., 2015). Knee hyper-extension, or genu recurvatum is a common gait issue, which is caused by poor muscle activation. It is observed in 40–60% of post-stroke patients (Cooper et al., 2012). Patients' pathological gait with severe myelopathy is also characterized with knee hyper-extension in the stance phase (Maezawa et al., 2001). This knee hypertension is developed to compensate their weaknesses in calf and quadriceps muscles, and remained even after 6 months when their velocity had improved (Mulroy et al., 2003). The knee hypertension is also prevalent among the obese population. The more extended leg may allow them to reduce vasti muscle activity and joint forces (DeVita and Hortobágyi, 2003). Their weakness in gluteus medius muscle may cause increased hip adduction during stance and require the stance leg to be extended more for the swing leg clearance (Haight et al., 2014). Once this kind of pathological gait is developed, the patients are more likely to develop knee hypertension and end up having a locomotive syndrome or requiring another surgical intervention (Bleyenheuft et al., 2010; Mao et al., 2015).

Prolonged hyper-extension or rapid extension may cause fatigue in the muscles responsible for stability, and chronic over tension causes weathering, degeneration and pain in the soft tissues that support the knee joint. The patients developed double knee action after the HAL intervention instead of resulting in knee hypertension, which led to smoother and closer to healthy gait.

EMG of VM, HAM, and GAS was analyzed to show that the gait improvement was achieved not only from a kinematical view, but also from a physiological perspective. The muscles that mainly control knee movement are HAM, GAS, and VM: HAM and GAS for knee flexion; VM for knee extension. The combination of those muscle's movement enables smooth knee action. The first knee action from initial contact to mid stance is important for shock absorption. HAM starts to regulate the rate of knee joint extension at late swing phase (87–100% of gait cycle) by efferent decelerating (Perry and Burnfield, 2010). There are several possible mechanisms to explain knee flexion limitation at the load response period. The complete lack of knee flexion in load-responsive period is often caused by an intentional compensatory movement to reduce the demand on quadriceps in case of weak quadriceps femoris (Mulroy et al., 2003). Possible causes of first knee action loss include weakness of the quadriceps femoris (Perry and Burnfield, 2010). Loss of first knee action reduces normal shock absorption and places less demand on the quadriceps.

In the acute phase, we observed EMG improvement in timing of muscle activity of HAM (Figure 4, Acute, HAM). HAM's improvement of timing in late swing phase is important for braking the swing, that suggests the preparation for flexion in initial contact. Due to the lack of accurate training, patients tend to use knee hypertension to compensate their muscle weakness. Without using HAM in swing phase, they keep extending their knees and tend to have difficulty landing with flexed knee at initial contact. After the HAL intervention, their breaking in swing phase got better.

In the chronic phase patients, there were no apparent changes in EMG of the three muscles after HAL intervention even though improvement of double knee action was observed in their joint angles. The joint angle analysis showed that the chronic patients had small first knee actions before the HAL intervention. Even they improved its flexion amount, this small improvement in joint angles were not obvious in EMG. It is also difficult to change their long-acquired compensatory gait motion over a comparatively short period of training once they got used to it. It has been shown that coordinated motor engrams need a tremendous amount of repeated training (Kottke et al., 1978). After formation of a pathological gait, the old engrams need to be inhibited, and it takes more time to relearn the movements. Longer periods of training, or early phase intervention might be required to modify their compensatory gait. Thus, it is important to re-learn healthy gait at the early stage of recovery with minimal compensation, particularly for those presents an asymmetric locomotion (Barbeau, 2003).

We observed improvements in walking speed, cadence, and stride length after the HAL training in both acute and chronic stage of postoperative OPLL patients. However, it is difficult to distinguish whether this recovery is achieved by the HAL intervention or solely in conjunction with patient's recovery of muscle strength. To clarify this, MMT scores of the participants before and after HAL intervention were compared. MMT scores of the acute patients showed improvement in hip flexor, knee extensor, and ankle dorsi flexor (Table 3). These improvements were mostly correlated with improvement in joint angle ROMs (Table 2). MMT scores of chronic patients showed improvement only in the hip flexor muscle (Table 5) which is correlated with significant improvement in ROM of hip joints (Table 4), but not in the other lower limb muscle groups even though their double knee action was improved. However, it is reported that the activity level of most muscles in normal gait of healthy people is equivalent to MMT 3 (Perry et al., 1986). Although muscle strength is necessary to achieve gait, muscle strength does not play the main role in gait improvement once basic necessary muscle strength is acquired. In this sense, learning to control timing and coordination of related muscles during gait is needed to achieve smooth and effective gait, besides the capability of maximum exertion of each single muscle.

As immediate change after HAL therapy, 10 m test before and after each 4th and 7th session was compared. Except the 7th HAL session of chronic patients, EMG of VM tended to decrease after HAL session during late to terminal phase, which indicates less knee extension. Also, EMG of GAS tended to decrease after HAL session except 7th acute patients, which indicates less ankle plantar flexion during late to terminal phase (Tables 7, 8). Both changes above were helpful to decrease hyper-knee extension. At the same time, their walking speed before and after the session did not change. This indicates that they were learning efficient gait without causing knee hypertension by HAL therapy. Considering also the results of MMT, it could be said that gait improvement by HAL includes improvement of coordinated gait control, not only muscle strengthening. It is noted that simple muscle strengthening exercises for a single or few muscles do not result in the acquisition of motor functions with functional reorganization of the brain (Plautz et al., 2000).

This study has limitations of not including a control group of OPLL patients undergoing conventional rehabilitation to be compared with patients after HAL intervention. Since OPLL is designated as an intractable disease in Japan, the number of patients is about 0.02% of the population (Matsunaga and Sakou, 2006), indicating difficulty establishing a control group. The number of severe paralysis patients who need assistance for sitting, standing, and walking is enrolled in our research, therefore the number of patients was even smaller. Clinical trial needs such patient control, which is left for future studies. Additionally, long-term prognosis was not investigated in our study. So far, a case report (Kubota et al., 2019) describes a patient with severe spinal cord disorder after OPLL who improved gait after HAL intervention, and the improved gait was maintained at the time of follow up assessment 1 year after the HAL intervention. To formalize a cohort study to track prognosis of the patients who underwent HAL treatment, it would be necessary to control the patients' daily activities to some extent in terms of the amount, frequency and strength of physical activities. It is left for future studies, where assessment of long-term prognosis using multivariate analysis after HAL intervention would be desired.

Muscle synergy analysis is reported as a useful tool for analyzing improved lateral symmetry of gait in stroke patients after HAL intervention (Tan et al., 2018). OPLL is not technically considered as causing lateral asymmetry, but patients usually develop asymmetrical gait since they tend to put more weight on a stronger leg and end up developing asymmetrical gait. In this sense, analysis of muscle synergy before and after HAL intervention may be a useful tool to evaluate muscle activity of OPLL patients (Kadone et al., 2020) after HAL intervention.

HAL is made to build a feedback loop like a normal voluntary movement, where movement is generated from voluntary neural and muscular activity and sensory input is provided. It was also shown that the awareness of voluntary actions is involved in the improvement of motor skills (Matsumiya, 2021). Though kinematic improvements were observed in this research, it is also important to study and clarify the neural mechanisms with which the HAL takes effect on the central nervous system. In future studies, radiological and neurophysiological assessments would help to further clarify these points.

Learning healthy gait instead of compensation gait is more desirable and it is expected in rehabilitation using medical exoskeletons which are focused on maximizing functional independence, balance, and restoring gait function. Those task-specific over-ground training is more helpful and supplement conventional rehabilitation (Esquenazi et al., 2017). The analysis presented in this study could help to establish indexes and scales to evaluate quality of gait so that the patients can regain healthier gait that can last long.

## 5. Conclusion

We measured and assessed the gait before and after the HAL intervention for postoperative OPLL patients with gait impairment in acute and chronic stage of post decompression operation. The patients gained faster walking speed, longer strides and smoother gait after the HAL intervention. ROM of hip, knee, and ankle joints have improved significantly in acute phase patients. All the joint angles except ankle joint have improved significantly in chronic patients. Importantly, double knee action of initial contact to loading response phase improved and knee hypertension was reduced, enabling the patients to gain closer to healthy gait. Also, all those improvement was not only made by muscle strengthening but also by learning coordinated gait control.

It is still true that conventional rehabilitation training is also efficient for patients. However, it would be difficult to repeat the exercises with the healthy gait pattern from the beginning to form a correct engram. It is possible for HAL to intervene from the acute stage even when walking is difficult without support, and to realize repetitive training of correct gait. During this period, it also includes muscle strengthening through natural recovery, but the difference is that it is not single muscle strengthening training, but functional motor learning of gait.

Staying in low ADL with less mobility or altered gait mechanics with increased pain, decreased muscle mass, proprioception deficits would cause other chronic diseases including osteoporosis, obesity, hyperglycemia and cardiopulmonary complications. The HAL can provide repeatable, standard, sustainable, and voluntary training and the current research reports the feasibility and effectiveness of HAL for functional recovery in rehabilitation use for multiple neurological disorders patients with gait disturbance after spinal cord diseases and cerebral vascular diseases.

## Data Availability Statement

The raw data supporting the conclusions of this article will be made available by the authors, without undue reservation.

## Ethics Statement

The studies involving human participants were reviewed and approved by The University of Tsukuba Hospital Ethics Committee. The patients/participants provided their written informed consent to participate in this study.

## Author Contributions

SE and HK collected, analyzed, and interpreted the data. SE wrote and drafted and HK edited the manuscript. SK administered HAL therapy and collected clinical scores. TA diagnosed and operated all the patients from acute group. YSh supported the HAL therapy. CT assisted the data analysis. KM, YH, and MK provided important comments on the planning and implementation of HAL treatment. YSa originally developed the robot suit HAL and conceived the idea of HAL therapy. KS provided essential insights for the analysis. MY operated all patients from chronic group, developed HAL therapy for OPLL patients, and organized the study. All authors made critical revisions of the manuscript and approved the final version.

## Conflict of Interest

YSa is the C.E.O., shareholder, and director of CYBERDYNE Inc., which produces the robot suit HAL. CYBERDYNE was not involved in the study design, data collection, analysis, writing, or submission of this article. The remaining authors declare that the research was conducted in the absence of any commercial or financial relationships that could be construed as a potential conflict of interest.
